# Restoring glenoid bone stock in failed anatomic shoulder arthroplasty: a cement-impression technique using femoral head allograft

**DOI:** 10.1016/j.xrrt.2026.100870

**Published:** 2026-08-31

**Authors:** Moritz Kraus, Mara Warnhoff, David Endell, Florian Freislederer, Asimina Lazaridou, Markus Scheibel

**Affiliations:** a Department of Shoulder and Elbow Surgery, Schulthess Clinic, Zurich, Switzerland; b Department of Traumatology, University Hospital Zürich, Switzerland; c Center for Musculoskeletal Surgery, Charité-Universitätsmedizin, Berlin, Germany; d Department of Anesthesiology, Brigham & Women's Hospital and Harvard Medical School, Boston, MA, USA

**Keywords:** Revision reverse shoulder arthroplasty, Glenoid bone loss, Femoral head allograft, Cement-impression technique, Glenoid reconstruction, Glenoid defect

Over the past decade, a significant decline in the proportion of anatomical prostheses has been observed in shoulder arthroplasty,^6^ while the total number of implantations increased by each year.^12^ Anatomic total shoulder arthroplasties (aTSAs) represent only about 35% of all shoulder joint replacements performed in Australia today, whereas this figure was over 60% in 2009.^2^ The lower revision rate of reverse shoulder arthroplasties (rTSAs) could be one of the reasons for this shift in trends, with 6% in rTSA vs. 13%^8^ in all aTSA. Glenoid component failure is a leading cause of revision in aTSA, due to a high incidence of glenoid loosening.^8^ Alongside secondary rotator cuff insufficiencies, glenoid loosening constitutes 24% of all complications in aTSA.^3^ After 3 years, 50% of patients display signs of glenoid lysis, escalating to up to 90% of patients after 10 years.^1^ The surgical management of glenoid loosening and concomitant glenoid defects in patients with shoulder arthroplasties is a challenging aspect in shoulder arthroplasty.^14^ Especially in all polyethylene glenoids, the lytic areas can progress to extensive glenoid destruction leading to the necessity of revisions in more than 50% of cases within 10 years of follow-up.^10^ Patients seeking revision for functional limitations and pain may require complex procedures, ideally conducted in specialized centers.^14^ Repairing extensive defects and performing revision arthroplasty in a single operation is often not feasible, leading to prolonged patient suffering and increased risk of surgical complications. Experienced surgeons should aim for a one-stage revision if low-grade infection as a cause of glenoid loosening is excluded, as recent results suggest comparable outcomes even in cases of periprosthetic infections.^11^ To optimally address these extensive defects, often exceeding several centimeters,^3^ we introduce a surgical technique that utilizes bone cement to create a negative impression of the defect. This imprint serves as a template for graft shaping, enabling an anatomically precise glenoid reconstruction and providing optimal conditions for graft integration. Even with pronounced defects, this approach facilitates sufficient anchorage for direct implantation of a revision TSA. For severe glenoid defects, metal-artifact–corrected 3-dimensional (3D) computed tomography with virtual implant removal^7^ enables precise defect characterization and pre-operative planning,^13^ thereby facilitating optimal bone grafting strategies and implant positioning.^15^

In this technical note, we describe the surgical technique used for glenoid reconstruction with a femoral head allograft during revision of a failed aTSA (Type Arthrex Eclipse with Pegged-PE-Glenoid) to an rTSA (Type Reversed II; Stryker, Portage, MI, USA).

## Case presentation

A 65-year-old male patient who presented with severe shoulder pain during physical activity and mild discomfort at rest, 10 years after receiving a primary aTSA (Arthrex Eclipse with cemented all Pegged-PE-Glenoid). Radiological findings indicated glenoid loosening (Figs. 1 and 2), which had progressed since the 5-year follow-up. The patient underwent a revision surgery with glenoid reconstruction using a femoral allograft, followed by the implantation of an rTSA. After 6 months and 2 years post-operatively, the patient demonstrated a satisfactory clinical outcome, with radiographic evidence of femoral head allograft incorporation, improved range of motion (ROM), and no signs of implant loosening.

**Figure 1:**
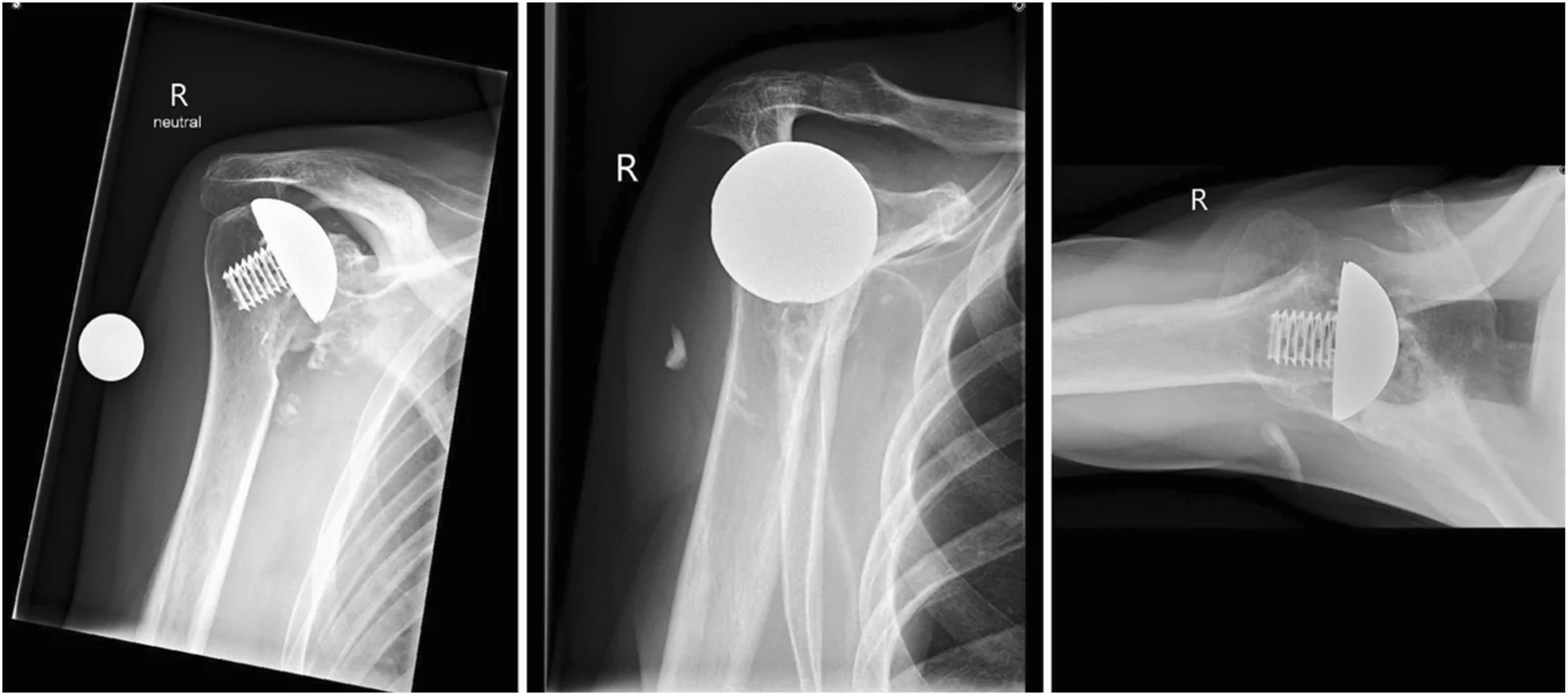
Ten-year X-ray follow-up after implantation of aTSA. *aTSA*, anatomic total shoulder arthroplasty.

**Figure 2:**
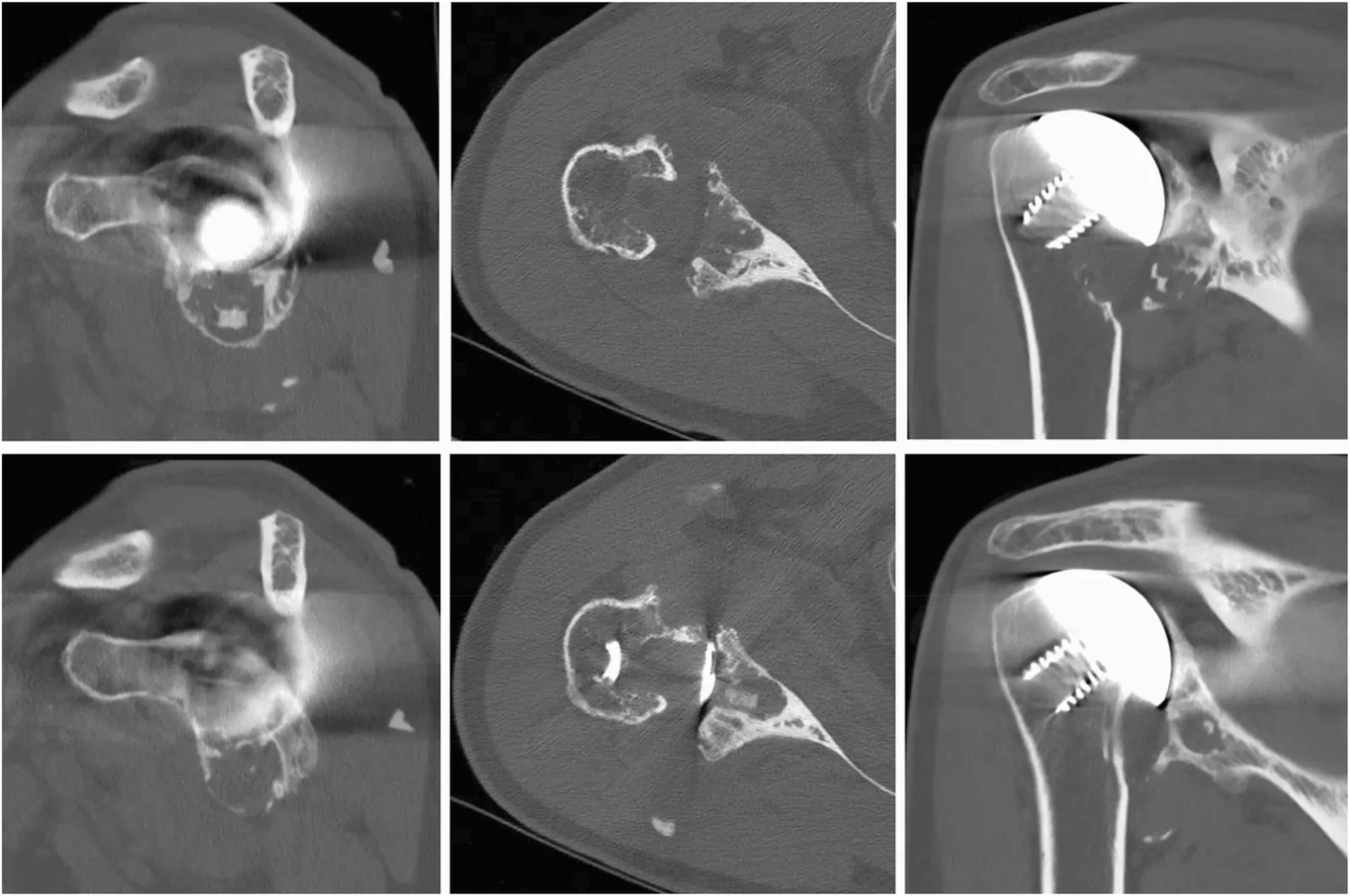
Computertomografie scan 10 years post-op after aTSA. *aTSA*, anatomic total shoulder arthroplasty.

### Examination findings

Upon examination, the patient’s right shoulder was externally unremarkable, and peripheral circulation, sensation, and movement of the right arm were intact. Active ROM showed anteversion of 160°, abduction of 140°, and external rotation of 60°. Passive ROM extended to 70°. The patient had the ability to perform the neck grip test and showed no signs of lag. The belly-press-test revealed 15° of movement. Bilateral abduction strength was 4 kg.

### Radiological findings

The X-ray of the right shoulder taken at the 10-year follow-up showed a known glenoid loosening with complete joint space obliteration, wear of the pegged-polyethylene inlay, and additional bone resorption in the calcar region (Fig. 2). There were prominent osteolysis both on the glenoid and humeral sides, with a 2 × 3cm defect in the calcar region, and heterotrophic ossifications medio-caudal of it. The pegged PE appeared completely depleted.

Due to the patient’s condition and radiological findings, a revision surgery was decided to reconstruct the glenoid using a femoral allograft and implant an rTSA.

Pre-operative 3D planning software was used to analyze the defect and simulate its reconstruction. A long-post baseplate was planned to secure the glenoid construct within the remaining native bone, ensuring that over 50% of the post was anchored in native bone. The patient was informed about a potential two-stage procedure, consisting of an initial glenoid reconstruction followed by a secondary implantation of revision rTSA components if primary stability could not be achieved during surgery.

### Surgical technique

The procedure was performed with the patient in the beach chair position. After standard sterile preparation and World Health Organization time-out, the skin was opened in the pre-existing scar. Subsequently, there is a reapplication of antiseptic agents to prevent any potential cutibacterium acnes infection. Following this the deltopectoral interval was exposed. The cephalic vein was preserved during lateral retraction. A subdeltoid release was performed, and adhesions were mobilized to improve exposure. The thinned subscapularis was detached and reinforced with 2 #2 fiber-wire sutures.

The humeral head was dislocated, and the humeral component was removed and sent for sonication. Partial metaphyseal osteolysis was noted. The humerus was prepared for implantation of a Reversed II humeral stem with 155° inclination.

Attention was then directed to the glenoid. Fragments of the fractured polyethylene component were removed, and a 270° capsular release was performed. A hypertrophic synovitis with intraosseous extension was débrided, and a type C3 glenoid defect was visible (Fig. 3).^4^

**Figure 3:**
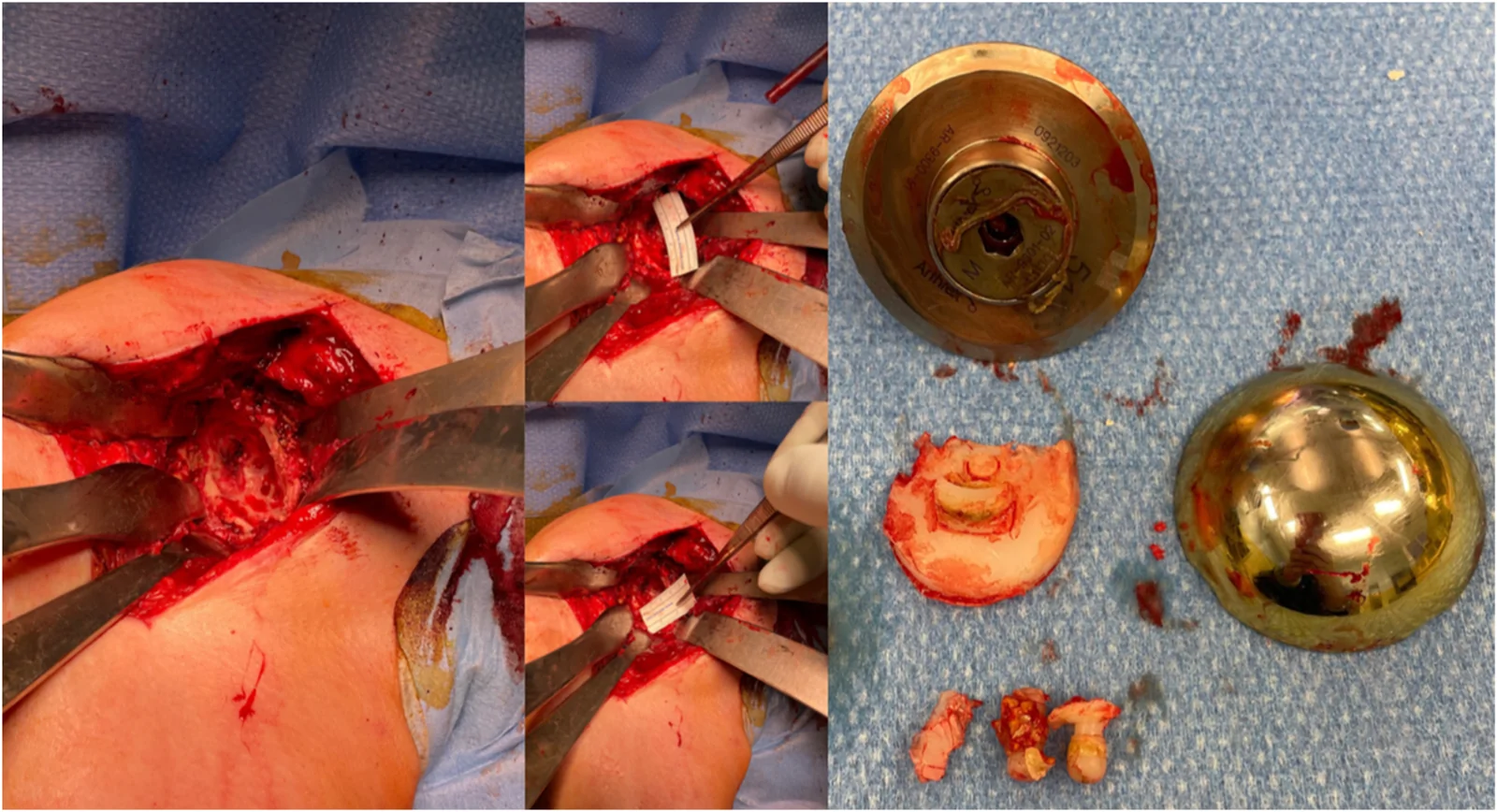
Intraoperative image of glenoid defect.

Bone cement was mixed and pressed into the glenoid defect to obtain an impression (Fig. 4). After curing for 7-8 minutes, the cement mold was removed and used as a template. A fresh-frozen femoral head allograft was then shaped to match the template. The allograft was initially contoured using an oscillating saw (System 8, Stryker, and Performance Series 6125-127-90 blades, Stryker) to match the gross dimensions of the cement template. Subsequently, fine-tuning of the graft geometry was performed using high-speed burr and rongeurs to achieve an accurate, press-fit adaptation into the contained glenoid defect on a sterile preparation table secured with different clamps by an assistant. A syringe of aspirated blood from the surgical field was used to impregnate the graft.

**Figure 4:**
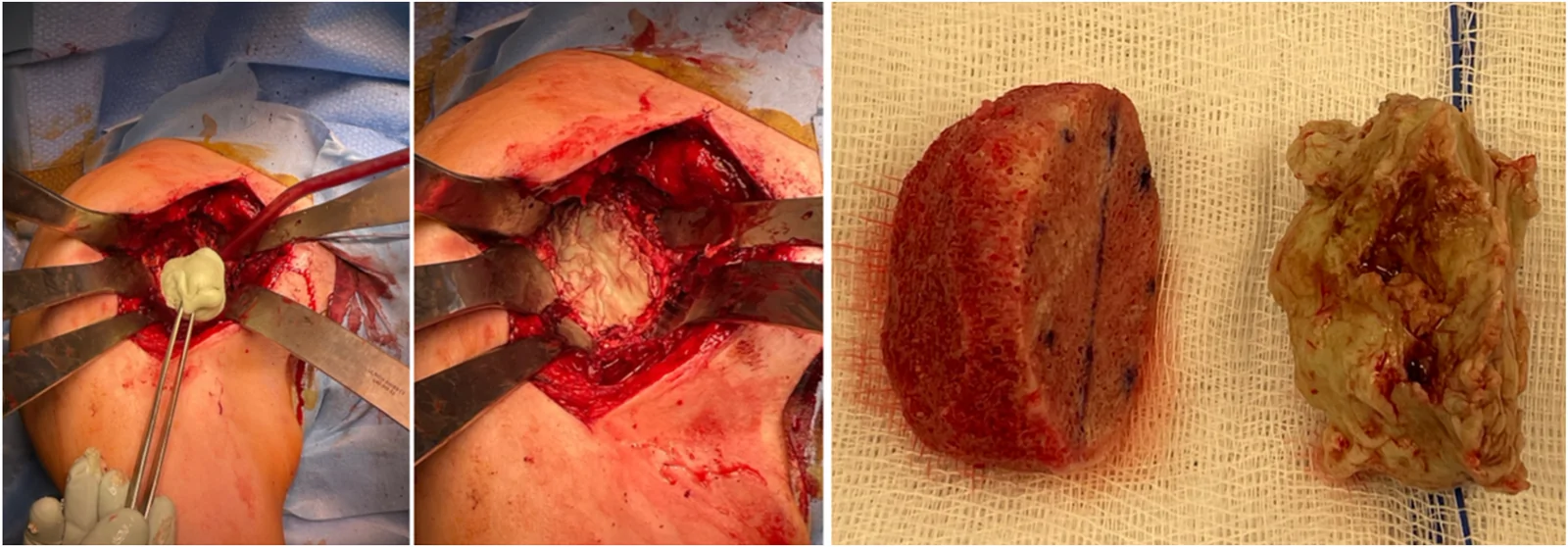
Cement impression and shaped bone graft.

The graft was impacted into the defect and temporarily secured with K-wires (Fig. 5). A long-post glenoid baseplate was placed and secured with 4 screws, allowing removal of the K-wires. Additional cancellous allograft bone chips were impacted to augment the construct. The structural graft was further contoured to achieve a restoration of the configuration of the anatomic glenoid contour. The procedure is summarized in Figure 6 and Table 1.

**Figure 5:**
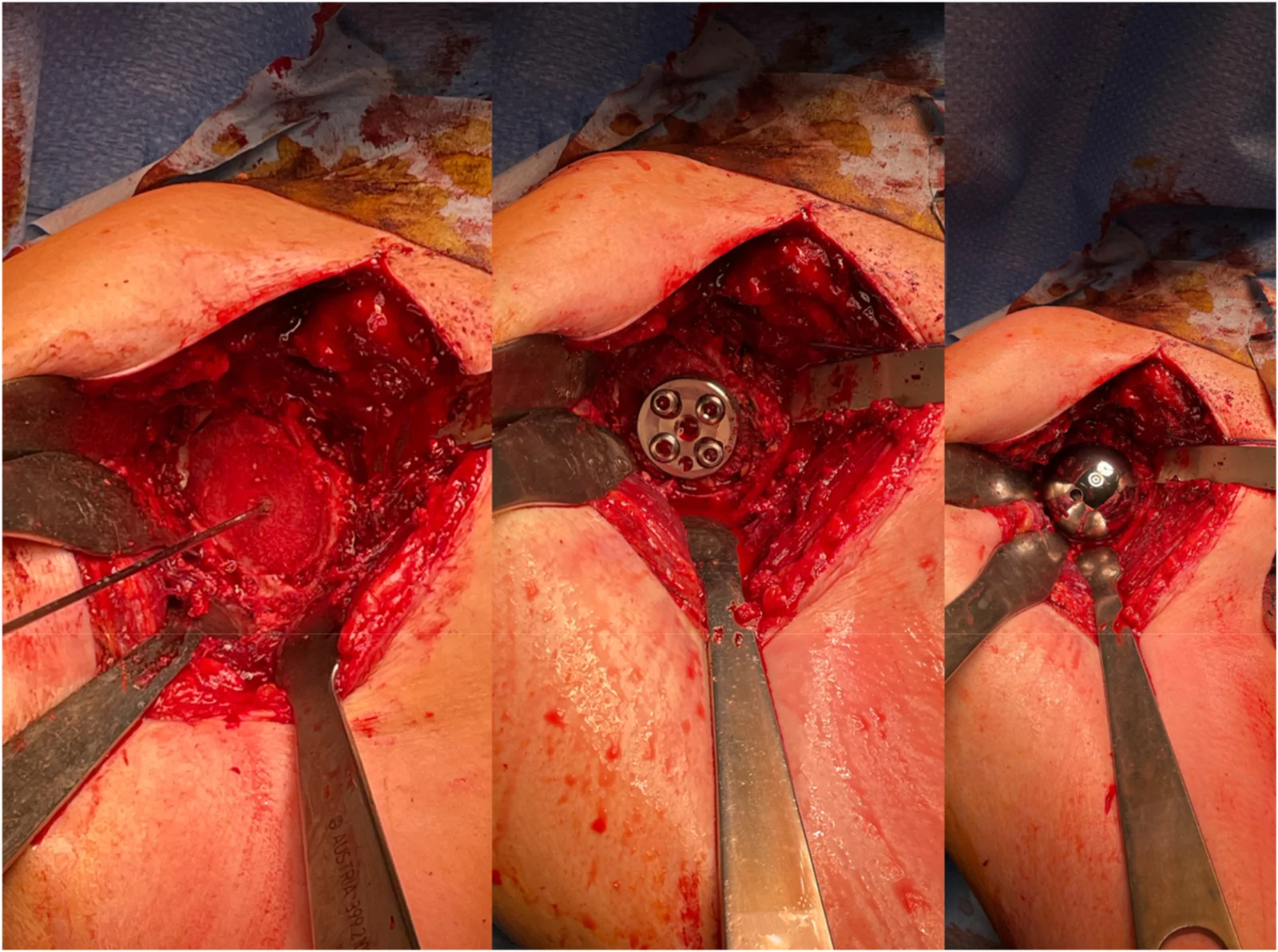
Impacted allograft (*left*), implanted baseplate (*middle*), implanted glenosphere (*right*).

**Figure 6:**
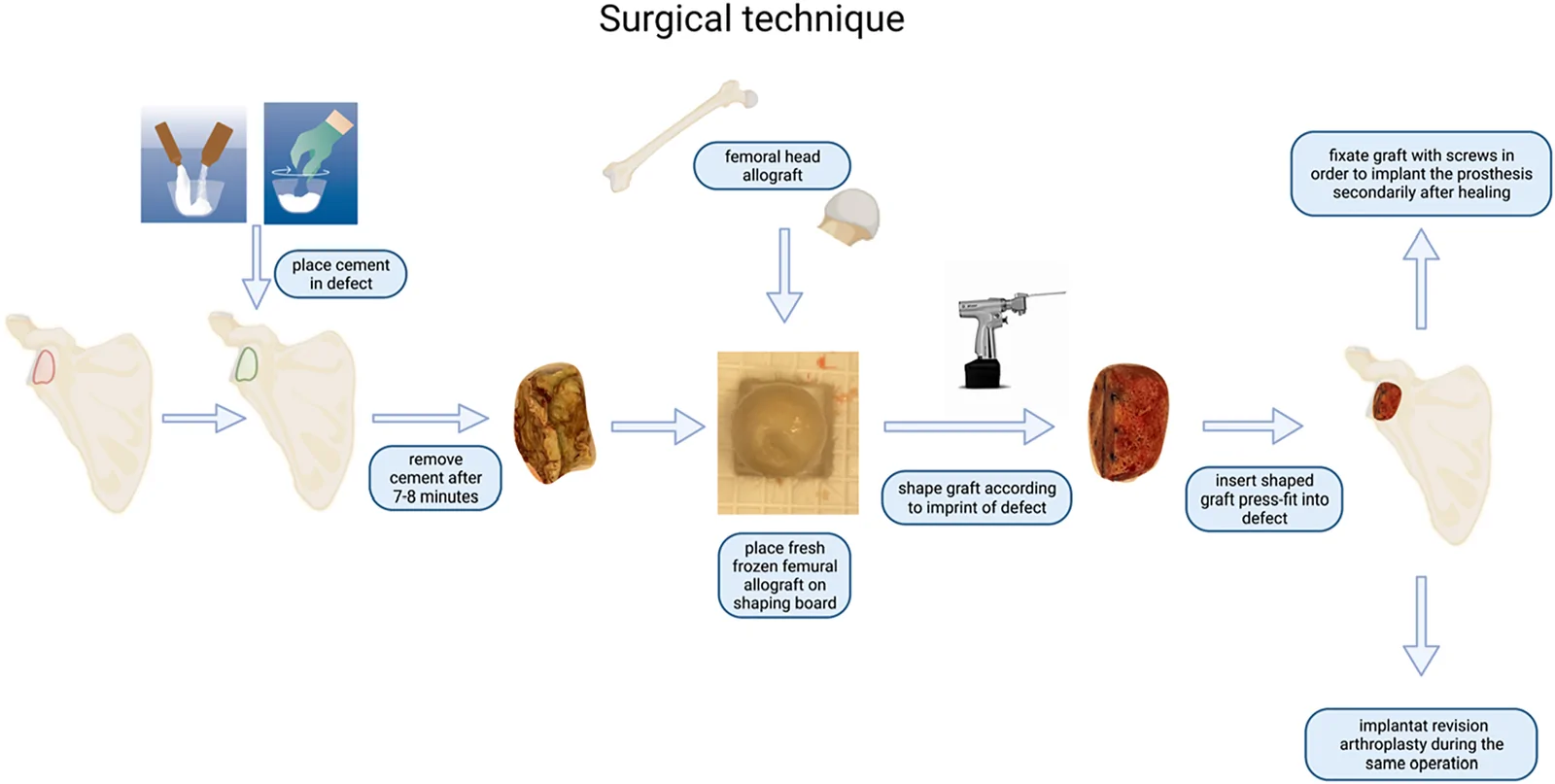
Graphical abstract of conducted technique.

**Table 1:** Surgical steps.

Step	Description
1. Patient positioning	Beach chair position
2. Surgical approach	Previous deltopectoral incision reopened; cephalic vein preserved; subdeltoid and capsular release performed.
3. Subscapularis management	Thinned tendon detached and reinforced with 2 #2 fiber-wire sutures.
4. Humeral preparation	Dislocation of humeral head; removal of humeral component; débridement and preparation for Reversed II humeral stem.
5. Glenoid exposure	Removal of polyethylene fragments; 270° release; synovectomy and débridement.
6. Impression technique	Bone cement molded into defect, left in place for 7-8 min, allowed to cure, and removed as a template.
7. Allograft preparation	Femoral head allograft shaped to match template; impregnated with autologous blood.
8. Graft implantation	Press-fit impaction; temporary K-wire fixation; cancellous chips added as needed.
9. Glenoid baseplate fixation	Long-post baseplate secured with 4 screws; K-wires removed.
10. Final implantation	Trial components tested; final components implanted cementlessly; fluoroscopic confirmation.
11. Closure	Irrigation, hemostasis, placement of drains, layered closure, and post-operative immobilization for 4 weeks

Following this, the rTSA (Type Reversed II) was implanted using a long-post baseplate (29 mm), a 42-mm +2-mm eccentric glenosphere, a cementless 100 × 15-mm humeral stem, a 42 metaphysis, and a +15-mm polyethylene inlay. Fluoroscopy confirmed correct alignment. The subscapularis tendon was reattached using 2 #2 FiberWires. The wound was irrigated, hemostasis secured, and 2 drains were placed. Layered wound closure and a compression dressing were applied, followed by immobilization in a MediArmFix (medi, Bayreuth, Germany) brace in internal rotation.

This approach provided stable reconstruction of the glenoid and allowed single-stage implantation of the rTSA.

### Post-operative follow-up

The patient was discharged with drains maintained for 48 hours and received post-operative antibiotics for 72 hours until the first results of the biopsies were confirmed. A restrictive post-operative protocol was initiated. Follow-up evaluations were conducted at 6 weeks (Fig. 7), 3 months, 6 months, and 24 months (Fig. 8).

**Figure 7:**
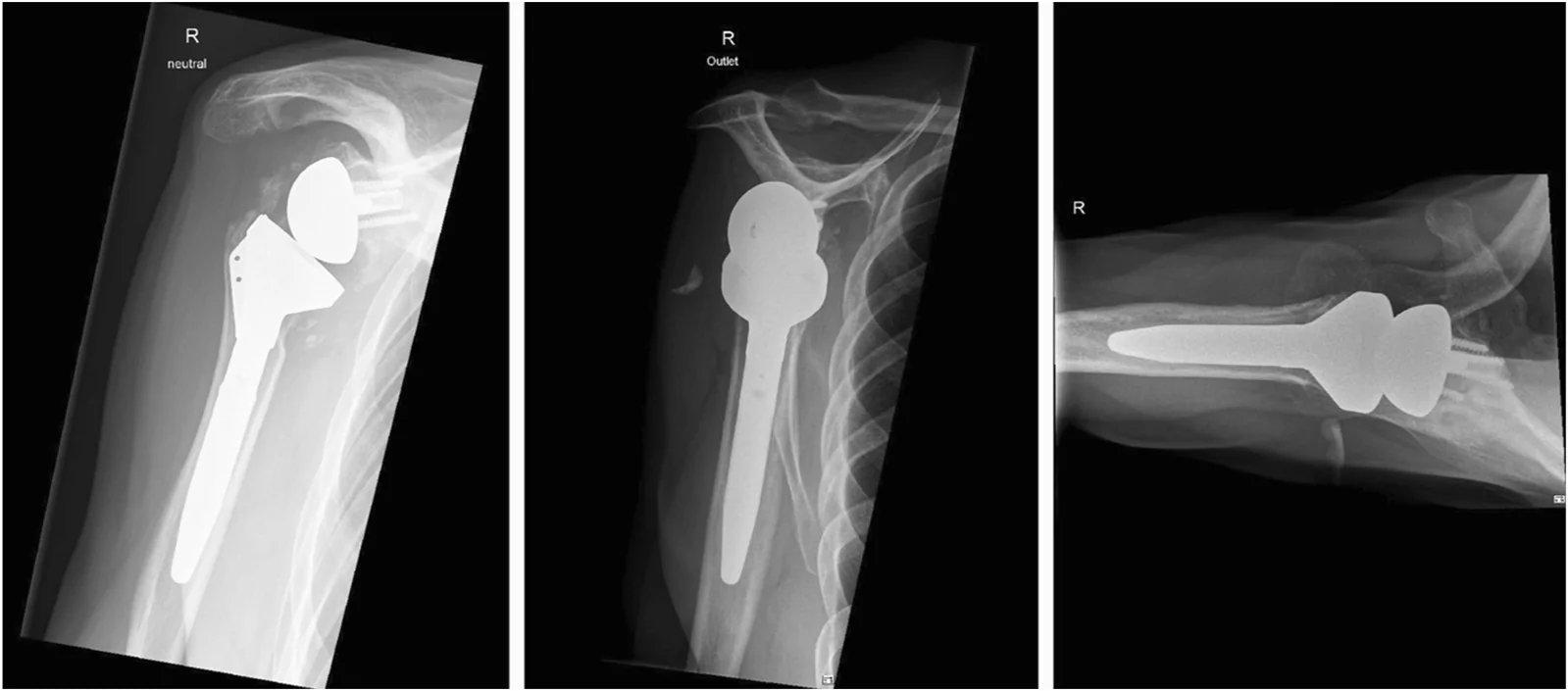
Six-week x-ray follow-up.

**Figure 8:**
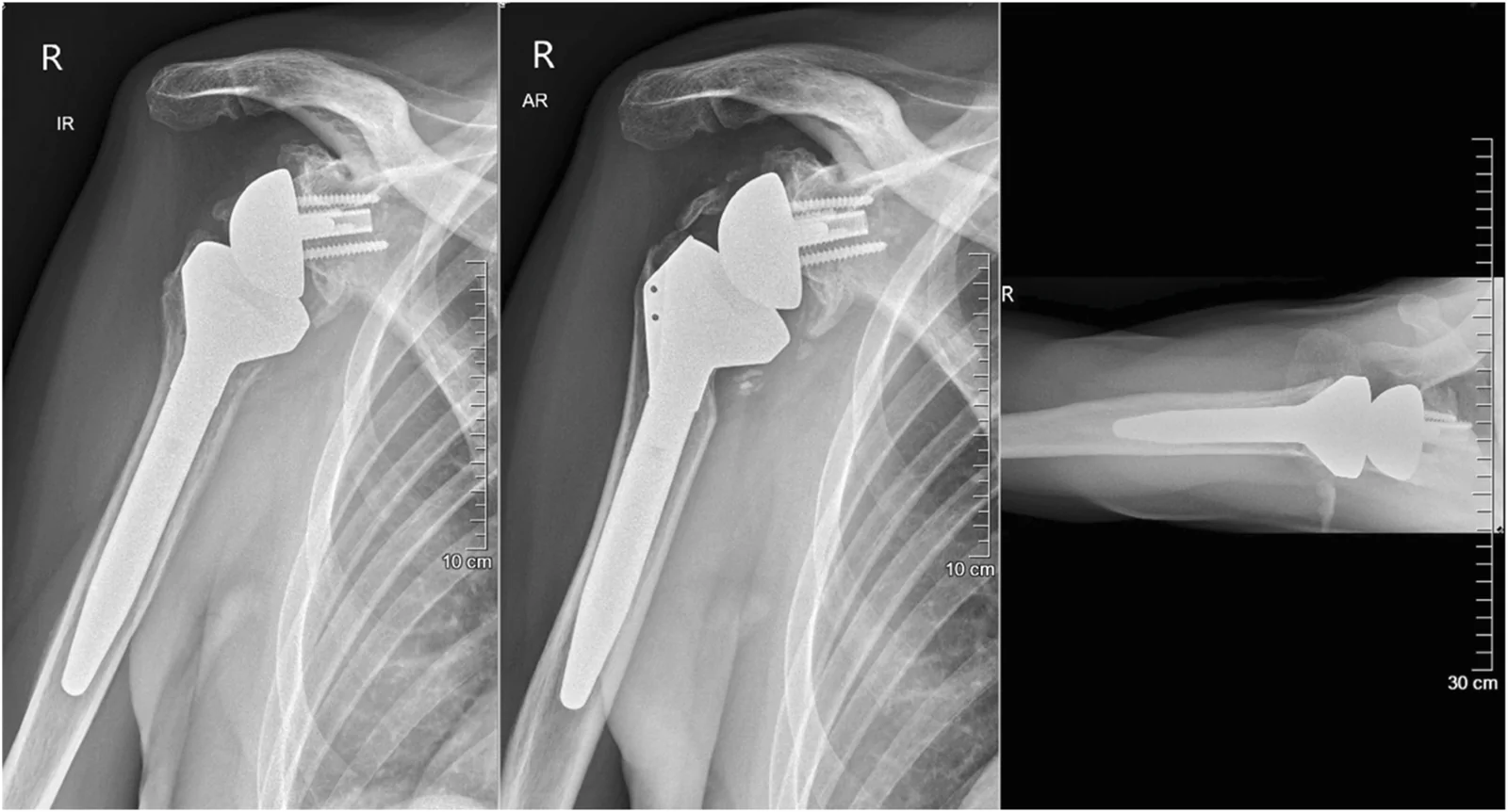
Twenty-four-month post-operative x-ray control.

### Six-week follow-up

Radiographs confirmed stable implant positioning without evidence of loosening. Clinical assessment demonstrated an actively assisted flexion of 90°, abduction of 60°, and 0° external rotation. The patient reported high satisfaction and had ceased all pain medication.

### Twelve-week follow-up

Imaging remained stable with no signs of loosening. Clinical ROM improved significantly to 150° flexion and 140° abduction. A specific medical training therapy for targeted muscle strengthening was initiated at this stage.

### Six-month follow-up visit

At the 6-month follow-up, the patient exhibited no clinical signs of complications. The radiological assessment revealed a centered to rTSA with minimal scapular notching. The patient expressed satisfaction with the results. A computertomografie scan confirmed successful consolidation of the femoral allograft and a stable glenosphere with no peri-implant osteolysis. Graft incorporation was defined as the presence of continuous trabecular bridging across the graft-host bone interface on the computertomografie scan, the absence of progressive radiolucent lines (>2 mm) around the graft, and the lack of graft fragmentation or hardware migration. Active ROM reached 170° flexion, 160° abduction, and 20° external rotation.

### Twenty-four-month follow-up

Final radiographs demonstrated a centered rTSA with stable, minor notching and no loosening. Functional outcomes were excellent: flexion 160°, abduction 160°, and a high external rotation of 70°. Abduction strength was measured at 8.5 kg. Sonography revealed fatty atrophy and chronic ruptures of the supraspinatus and infraspinatus tendons, while the teres minor remained normotrophic. Overall, the patient demonstrated a satisfactory clinical outcome with no specific limitations.

## Discussion

Single-stage revision to rTSA using structural femoral head allografting for massive contained glenoid defects achieved stable graft incorporation and excellent functional outcomes at the 24-month follow-up. This technique represents an approach for managing severe glenoid bone loss, enabling fixation of revision implants through intraoperative graft molding and impaction.

In this case, the structural allograft and its adaptation to defect morphology showed radiographic incorporation in single-stage revision rTSA.

Thorough débridement and careful preparation of the contained defect were essential to optimize conditions for graft integration. When an anatomic rim is preserved (C3),^4^ this method allows accurate restoration of glenoid geometry and may reduce the risk of recurrent instability.

Non-contained glenoid defects may necessitate alternative reconstructive methods, such as 2-stage procedures or patient-specific augments.^5^ The cement-impression technique is suitable for contained, cavitary defects (type C3) with a preserved bony rim to support the graft. It is contraindicated in cases of significant segmental loss of the glenoid vault (type E/C4) or active high-grade periprosthetic joint infection, where reliable negative impressions cannot be obtained.

The decision to implant this model of a Reversed II arthroplasty was based on its medialized design and reduced glenoid loading, which help mitigate implant loosening in cases with compromised bone stock. The chosen glenosphere configuration provided additional stability and optimized joint biomechanics, supporting early mobilization and functional recovery.

Post-operative rehabilitation remains a critical determinant of long-term success. Structured protocols and patient adherence are essential to preserve joint stability and maximize functional gains.

The innovation of the approach lies in the intraoperative creation of a patient-specific defect template, distinguishing it from traditional methods that rely on visual estimation for graft contouring. While augmented glenoid baseplates can address specific wear patterns, they may not effectively manage large, cavitary C3 defects seen in revision of an aTSA with significant glenoid lysis. This technique offers a substantial alternative in situations where patient-specific implants are unavailable or in cases of unexpected glenoid bone loss, providing a prompt, cost-effective solution that avoids the delays of custom manufacturing while still offering a degree of anatomical customization tailored to the specific morphology of the defect.

Patient-specific implants represent a promising option for the management of severe and complex glenoid defects, as they allow individualized reconstruction based on patient-specific anatomy and may facilitate restoration of glenoid bone stock and implant positioning. However, important practical and logistical limitations still exist. Production of a custom implant often requires multiple weeks from imaging to manufacturing, which can delay surgery, during which time the bone defect may progress.^9^ Moreover, the additional cost and need for specialized manufacturing limit widespread use, particularly in urgent revision settings.^5^ In contrast, the presented technique using a structural allograft and intraoperative “impression” molding allows personalized graft shaping without prolonged waiting periods or the higher cost of customized implants. This method enables timely, cost-efficient reconstruction with a graft tailored to the patient's defect. Looking forward, a hybrid approach may become viable: pre-operative 3D printing of a negative plastic model of the defect could be done in-house within days, sterilized, and used intraoperatively as a template for graft shaping. Such an approach would combine the benefits of 3D planning and customization with the flexibility and immediacy of bone grafting, offering an efficient alternative to full custom implants.

### Limitations

The impression technique depends on the presence of an intact circumferential glenoid rim, which limits its applicability in non-contained defects or severe vault destruction. Furthermore, precise timing and management of the cement impression, including prompt removal to avoid complications and accounting for cement expansion during curing, are essential. As this report describes a single case, further studies with larger patient cohorts are required to validate the long-term clinical outcomes and generalizability of the cement-impression technique in revision rTSA.

## Conclusion

This technique enables anatomical reconstruction of glenoid defects in cases with an intact circumferential rim, facilitating accurate 3-dimensional defect replication and incorporation of the allogeneic bone graft. Precise graft adaptation may contribute to initial implant stability while allowing single-stage revision surgery. Further refinement and interdisciplinary investigation may support broader clinical application of this approach.

## Disclaimers

Funding: No funding was disclosed by the authors.

Conflicts of interest: Markus Scheibel and Florian Freislederer serve as consultants for and receive royalties from Stryker, Inc. Any additional authors, their immediate families, and any research foundations with which they are affiliated have not received any financial payments or other benefits from any commercial entity related to the subject of this article.
